# Antibacterial and Antibiofilm Activities of Tryptoquivalines and Meroditerpenes Isolated from the Marine-Derived Fungi *Neosartorya paulistensis*, *N. laciniosa*, *N. tsunodae*, and the Soil Fungi *N. fischeri* and *N. siamensis*

**DOI:** 10.3390/md12020822

**Published:** 2014-01-28

**Authors:** Nelson M. Gomes, Lucinda J. Bessa, Suradet Buttachon, Paulo M. Costa, Jamrearn Buaruang, Tida Dethoup, Artur M.S. Silva, Anake Kijjoa

**Affiliations:** 1 ICBAS—Instituto de Ciências Biomédicas de Abel Salazar, Rua de Jorge Viterbo Ferreira, 228, Porto 4050-313, Portugal; E-Mails: ngomes@ciimar.up.pt (N.M.G.); lbessa@ciimar.up.pt (L.J.B.); nokrari_209@hotmail.com (S.B.); pmcosta@icbas.up.pt (P.M.C.); 2 Interdisciplinary Centre of Marine and Environmental Research (CIIMAR), Universidade do Porto, Rua dos Bragas 289, Porto 4050-123, Portugal; 3 Division of Environmental Science, Faculty of Science, Ramkhamhaeng University, Bangkok 10240, Thailand; E-Mail: jbuaruang@ru.mail.go.th; 4 Department of Plant Pathology, Faculty of Agriculture, Kasetsart University, Bangkok 10240, Thailand; E-Mail: agrtdd@ku.ac.th; 5 Departamento de Química, Universidade de Aveiro, Aveiro 4810-1933, Portugal; E-Mail: artur.silva@ua.pt

**Keywords:** antibacterial, antibiofilm, multidrug-resistant, tryptoquivalines, meroditerpenes, *Neosartorya*, marine-derived fungi

## Abstract

A new meroditerpene, sartorypyrone C (**5**), was isolated, together with the known tryptoquivalines l (**1a**), H (**1b**), F (**1c**), 3′-(4-oxoquinazolin-3-yl) spiro[1*H*-indole-3,5′]-2,2′-dione (**2**) and 4(3*H*)-quinazolinone (**3**), from the culture of the marine sponge-associated fungus *Neosartorya paulistensis* (KUFC 7897), while reexamination of the fractions remaining from a previous study of the culture of the diseased coral-derived fungus *N. laciniosa* (KUFC 7896) led to isolation of a new tryptoquivaline derivative tryptoquivaline T (**1d**). Compounds **1a**–**d**, **2**, **3**, and **5**, together with aszonapyrones A (**4a**) and B (**4b**), chevalones B (**6**) and C (**7a**), sartorypyrones B (**7b**) and A (**8**), were tested for their antibacterial activity against four reference strains (*Staphylococcus aureus*, *Bacillus subtilis*, *Escherichia coli*, and *Pseudomonas aeruginosa*), as well as the environmental multidrug-resistant isolates. Only aszonapyrone A (**4a**) and sartorypyrone A (**8**) exhibited significant antibacterial activity as well as synergism with antibiotics against the Gram-positive multidrug-resistant strains. Antibiofilm assays of aszonapyrone A (**4a**) and sartorypyrone A (**8**) showed that practically no biofilm was formed in the presence of their 2× MIC and MIC. However, the presence of a sub-inhibitory concentration of ½ MIC of **4a** and **8 ** was found to increase the biofilm production in both reference strain and the multidrug-resistant isolates of *S. aureus.*

## 1. Introduction

Infectious diseases are leading health problems with high morbidity and mortality in the developing countries. Although the introduction of penicillin and other antibiotics ushered in an era of effective treatment of microbial infection, their overuse has caused acquired resistance of pathogens to antimicrobial agents. Since the mid-1970s, resistance to antimicrobial agents has become an escalating problem [1]. In the last 30 years, treatment of infections caused by Gram-positive bacteria has been more problematic than ever, with infections being caused by multidrug-resistant organisms, particularly methicillin-resistant staphylococci, penicillin- and erythromycin-resistant pneumococci, and vancomycin-resistant enterococci [2]. The development of resistance to multiple drugs is therefore a worldwide problem in the treatment of these infectious diseases caused by clinically relevant pathogenic microorganisms and must be approached in a large variety of strategies [3]. Although there is a continuing effort in the pharmaceutical industry to develop new antimicrobial agents for the treatment of resistant infections, pursuing new antibiotic drugs is still a fair and necessary strategy to combat the multidrug-resistant bacteria that are spreading both in the community and clinical setting [4]. Since the marine environment is a prolific source of bioactive compounds with extraordinary chemical and biological diversity [5,6,7], it has become a potential target in the search for new antibiotics. Specifically, the marine-derived fungi which have been reported as producers of bioactive metabolites with antiviral [8,9], antitumor [10,11] and antibacterial [12,13,14] activities. In the pursuit for bioactive secondary metabolites produced by marine and soil fungi of the genus *Neosartorya*, we have recently reported isolation and structure elucidation of sartorypyrone A (**8**), aszonapyrone A (**4a**), aszonalenin, acetylaszonalenin, 1-formyl-5-hydroxyaszonalenin and 13-oxofumitremorgin B, from the culture of the soil fungus *Neosartorya fischeri* (KUFC 6344), sartorypyrone B (**7b**) from the marine sponge-associated fungus *N. tsunodae*, as well as aszonapyrone A (**4a**), aszonapyrone B (**4b**), tryptoquivaline L (**1a**) and 3′-(4-oxoquinazolin-3-yl) spiro[1*H*-indole-3,5′-oxolane]-2,2′-dione (**2**) from *N. laciniosa* isolated from a diseased coral [15].

**Figure 1:**
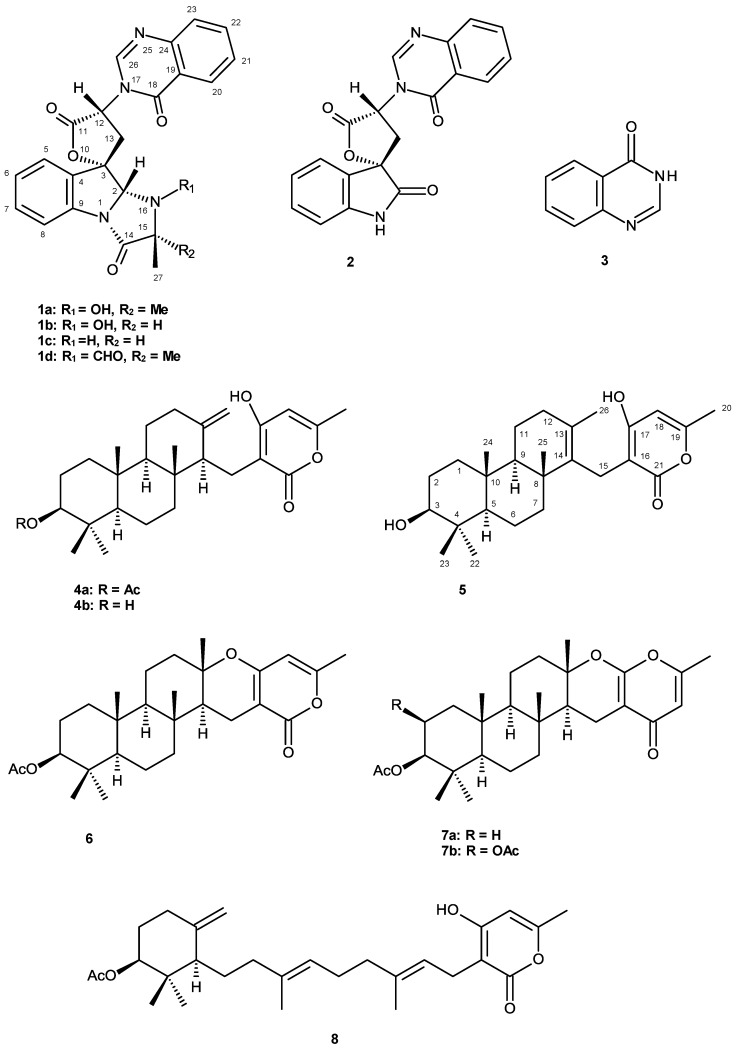
Secondary metabolites from *Neosartorya paulistensis*, *N. laciniosa*, *N. siamensis*, *N. tsunodae* and *N. fischeri*.

Examination of a collection of *N. paulistensis* (KUFC 7897), isolated from the marine sponge *Chondrilla australiensis*, collected from the Gulf of Thailand, resulted in isolation of a new aszonapyrone analogue which we have named sartorypyrone C (**5**), in addition to five known metabolites including tryptoquivalines L (**1a**), H (**1b**), F (**1c**), 3′-(4-oxoquinazolin-3-yl) spiro[1*H*-indole-3,5′-oxolane]-2, 2′-dione (**2**) and 4(3*H*)-quinazolinone (**3**) (Figure 1). Reexamination of the column fractions left over from our previous work of *N. laciniosa* (KUFC 7896) [15] led to isolation of a new tryptoquivaline analogue, tryptoquivaline T (**1d**), whereas reexamination of the nonpolar fractions from the column chromatography of *N. siamensis* (KUFC 6349) [16] furnished chevalone B (**6**) and chevalone C (**7a**) (Figure 1). The isolated compounds were evaluated, together with sartorypyrone B (**7b**) previously isolated from *N. tsunodae* and sartorypyrone A (**8**) (Figure 1) previously isolated from *N. fischeri*, for antibacterial activity against the Gram-positive (*Staphylococcus aureus* ATCC 25923 and *Bacillus subtilis* ATCC 6633) and Gram-negative (*Escherichia coli* ATCC 25922 and *Pseudomonas aeruginosa* ATCC 27853) bacteria, as well as multidrug-resistant isolates from the environment. The potential synergism between these fungal metabolites and antibiotics was evaluated against multidrug-resistant bacteria, methicillin-resistant *S. aureus* (MRSA) and vancomycin-resistant Enterococci (VRE). Since aszonapyrone A (**4a**) and sartorypyrone A (**8**) exhibited interesting antibacterial activity against both Gram-negative reference strains and the environmental multidrug-resistant isolates, their capacity to inhibit biofilm formation was also studied.

**Table 1:** ^1^H and ^13^C NMR (DMSO, 300.13 and 75.47 MHz) and HMBC assignment for tryptoquivaline T (**1d**).

Position	δ_C_, Type	δ_H_, (*J* in Hz)	COSY	HMBC
1	---	---		
2	81.0, CH	6.10, s	H-29	C-3, 13, 14
3	84.2, C	---		
4	133.5,C	---		
5	126.8, CH	8.04, dd (8.0, 1.0)	H-6	C-3, 7, 9
6	126.7, CH	7.46, ddd (8.0, 8.0, 1.0)	H-5	C-4, 8
7	131.8, CH	7.61, ddd (8.0, 8.0, 1.0)	H-6, 8	C-5, 9
8	116.4, CH	7.56, ddd (8.0, 1.0)	H-7	C-4, 6
9	138.4, C	---		
11	171.1, CO	---		
12	57.7, CH	5.47, dd (10.7, 8.6)	H-13	C-11, 18
13a	33.8, CH_2_	3.30, dd (14.0, 8.6)	H-12, 13b	C-2, 3, 4
b		3.40, dd (14.0, 10.7)	H-12, 13a	C-2, 3, 4
14	172.5, CO	---		
15	64.1, C	---		
18	160.0, CO	---		
19	121.4, C	---		
20	126.2, CH	8.26, d (8.0, 1.0)	H-21	C-18, 22, 24
21	127.6, CH	7.63, ddd (8.0, 8.0, 1.0)	H-20, 22	C-19, 23
22	135.1, CH	7.93, ddd (8.0, 8.0, 1.0)	H-21, 23	C-20, 24
23	127.4, CH	7.76, d (8.0)	H-22	C-19, 21
24	147.8, C	---		
26	148.1, CH	8.62, s		C-12, 18, 24
27	26.6, CH_3_	1.72, s		C-14, 15, 28
28	25.5, CH_3_	1.55, s		C-14, 15, 27
29	162.3, CHO	8.73, d (0.9)	H-2	C-2

## 2. Results and Discussion

Compound **1d **was isolated as white solid, and its molecular formula C_24_H_20_N_4_O_5_ was established on the basis of the (+)-HRESIMS *m/z* 445.1512 [M + H]^+^, indicating 17 degrees of unsaturation. The IR spectrum showed absorption bands for aromatic (3010, 1582, 1450 cm^−1^) and carbonyls of ester/amide groups (1700 cm^−1^). The general features of the ^1^H and ^13^C spectra of **1d** (Supplementary Information, Figures S1 and S2) closely resembled those of tryptoquivaline L (**1a**). The ^13^C NMR, DEPT and HSQC spectra (Table 1) revealed three amide/ester carbonyls (δ_C_ 172.5, 171.1 and 160.0), one *N*-formyl (δ_C_ 162.3), four quaternary sp^2^ (δ_C_ 147.8, 138.4, 133.5, 121.4), nine methine sp^2^ (δ_C_ 148.1, 135.1, 131.8, 127.6, 127.4, 126.8, 126.7, 126.2, 116.4), two quaternary sp^3^ (δ_C_ 84.2 and 64.1), two methine sp^3^ (δ_C_ 81.0 and 57.7), one methylene sp^3^ (δ_C_ 33.8) and two methyl (δ_C_ 26.6 and 25.5) carbons.

Analysis of the ^1^H, ^13^C NMR, HSQC, COSY and HMBC (Table 1) revealed the presence of the *N*-substituted quinazolin-4-one and the 6-5-5 *gem*-dimethyl imidazoindole ring systems which were connected via a five membered spirolactone as in tryptoquivaline l [16]. However, the only difference between **1d **and tryptoquivaline l (**1a**) is the presence of the formyl group on N-16 of the *gem*-dimethyl imidazoindole moiety in the former and a hydroxyl group in the latter. On the other hand, the structure of **1d** differs from that of tryptoquivaline O, previously reported from *N. siamensis* by Buttachon *et al.* [16], in that there are two methyl groups on C-15 of the imidazoindole ring in the former instead of one methyl group in the latter. The assignments of the proton and carbon chemical shifts for CH_3_-27 and CH_3_-28 were based on the NOESY correlation between the signals of H-2 (δ_H_ 6.10, s) and CH_3_-27(δ_H_ 1.72, s). Thus, **1d** is a new tryptoquivaline analogue which we have named tryptoquivaline T. Since the chemical shift values of H-2 and H-12 of tryptoquivaline T (**1d**) are similar of those of the corresponding protons of tryptoquivaline O, we assume that the stereochemistry of tryptoquivaline T (**1d**) is the same as that of tryptoquivaline O, *i.e.*, C-2*S*, C-3*S* and C-12*R*. This assumption was also supported by the negative value of the rotation tryptoquivaline T (**1d**).

Compound **5** was also isolated as white solid (mp, 200–202 °C) and its molecular formula C_26_H_38_O_4_ was established on the basis of the (+)-HRESIMS *m/z* 415.2836 [M + H]^+^ (calculated 415.2848), indicating eight degrees of unsaturation. The IR spectrum showed absorption bands for hydroxyl (3445 cm^−1^), conjugated ester carbonyl (1668 cm^−1^) and olefin (1649, 1636 cm^−1^) groups. The ^13^C NMR, DEPT and HSQC spectra (Table 2) exhibited the signals of one conjugated ester carbonyl (δ_C_ 164.6), five quaternary sp^2^ (δ_C_ 164.3, 159.3, 136.6, 126.2. 101.4), one methine sp^2 ^(δ_C_ 99.9), three quaternary sp^3^ (δ_C_ 38.8, 38.4 and 36.8), one oxymethine sp^3^ (δ_C_ 76.9), two methine sp^3^ (δ_C_ 56.0, 54.8), seven methylene sp^3^ (δ_C_ 38.0, 37.9, 34.2, 27.1, 22.1, 18.2, 17.7), and six methyl (δ_C_ 28.1, 21.2, 20.3, 19.2, 16.3 and 15.7) carbons. Except for the presence of one more methyl group instead of an exocyclic methylene group, and a tetra-substituted double bond (δ_C_ 126.2 and 136.6), the ^1^H and ^13^C data (Table 2, Supplementary Information, Figures S3 and S4) revealed the existence of the perhydrophenanthrene moiety connected to the 4-hydroxy-6-methyl-2*H*-pyran-2-one portion through the methylene group, similar to those of aszonapyrone B (**4b**) [15]. That the double bond in the perhydrophenanthrene moiety was on C-13 and C-14 was supported by the HMBC correlations of H_3_-26 (δ_H_ 1.55) to C-12 (δ_C_ 34.2), C-13 (δ_C_ 126.2), C-14 (δ_C_ 136.6); H-15 (δ_H_ 3.02, brs) to C-8 (δ_C _38.8), C-13, C-14, C-16 (δ_C _101.4), C-17 (δ_C _164.3) and C-21 (δ_C _164.6). That the hydroxyl group on C-3 was β is supported by the chemical shift value (δ_C_ 15.7) of the C-4 axial methyl (CH_3_-23) which suffered a γ-*gauche* interaction [17]. Thus, compound **5** is a new analogue of aszonapyrones which we have named sartorypyrone C.

**Table 2:** ^1^H and ^13^C NMR (DMSO, 300.13 and 75.47 MHz) and HMBC assignment for sartorypyrone C (**5**).

Position	δ_C_, Type	δ_H_, (*J* in Hz)	COSY	HMBC
1	37.9, CH_2_	1.65, m	H-2	
2	27.1, CH_2_	1.46, m	H-1, 3	
3	76.9, CH	2.97, m	H-2	
4	38.4, C	---	---	
5	54.8, CH	0.67, brd (9.6)	H-6	
6	17.7, CH_2_	1.35, m	H-5, 7	
7	38.0, CH_2_	1.96, m	H-6	
8	38.8, C	--		
9	56.0, CH	0.97, brd (11.4)	H-11	CH_3_-24
10	36.8, C	---		
11	18.2, CH_2_	1.46, m	H-9, 12	
12	34.2, CH_2_	1.94, m	H-11	
13	126.2, C	---	---	
14	136.6, C	---	---	
15	22.1, CH2	3.02, brs	---	C-8, 13, 14, 16, 17, 21
16	101.4, C	---	---	
17	164.3, C	---	---	
18	99.9, CH	5.90, s		CH_3_-20, C-1617, 19, 20
19	159.3, C	---	---	
20	19.2, CH_3_	2.12, s	H-18	C-18, 19
21	164.6, C	---	---	C-3, 4, 5, 23
22	28.1, CH_3_	0.86, s	---	C-3, 4, 5, 22
23	15.7, CH_3_	0.66, s	---	
24	16.3, CH_3_	0.77, s	---	C-1, 5, 9, 10
25	21.2, CH_3_	0.89, s	---	C-8, 9, 14
26	20.3, CH_3_	1.55, s	---	C-12, 13, 14

Compounds **1**–**8** (Figure 1) were tested for their antibacterial activity against bacterial reference strains and environmental multidrug-resistant isolates, and their MIC and MBC (when determined) values are shown in Table 3A. It is interesting to note that neither of the indole alkaloids (**1a**–**d**, **2**) exhibited relevant antibacterial activity. However, within the meroditerpene group, only aszonapyrone A (**4a**) and sartorypyrone A (**8**) presented significant MIC values against Gram-positive bacteria. Aszonapyrone A (**4a**) showed the MIC values of 8 µg/mL against *S. aureus* ATCC 25923 and *B. subtilis* ATCC 6633, while sartorypyrone A (**8**) showed the MIC values of 32 and 64 µg/mL, respectively, against the same reference strains. Based on these results, the MIC values of these two compounds were further determined against Gram-positive multidrug-resistant strains. While aszonapyrone A (**4a**) was found to be active against both *S. aureus* MRSA and *Enterococcus* spp. VRE isolates, sartorypyrone A (**8**) did not show any inhibition on the growth of *Enterococcus* spp. VRE isolates in the range of concentrations tested (Table 3B). MBC values were only achieved for aszonapyrone A (**4a**) against Gram-positive reference strains, and since sartorypyrone A (**8**) did not exhibit any bactericidal effect against any strain, its MBC values could not be determined.

**Table 3:** Antimicrobial activity, expressed in µg/mL of **1**–**8** against references strains (**A**) and of **4a** and **8** against multidrug-resistant isolates (**B**).

**(A)**								
**Compounds**	***S. aureus* ATCC 25923**		***P. aeruginosa* ATCC 27853**		***B. subtilis* ATCC 6633**		***E. coli* ATCC 25922**	
	**MIC**	**MBC**	**MIC**	**MBC**	**MIC**	**MBC**	**MIC**	**MBC**
**1a**	128	−	128	256	128	−	128	−
**1b**	128	−	128	256	128	−	128	−
**1c**	128	−	128	128	128	−	128	−
**1d**	−	−	128	−	128	−	128	−
**2**	256	−	128	256	128	−	128	−
**3**	128	−	128	256	128	−	128	−
**4a**	8	64	128	256	8	16	128	−
**4b**	256	−	128	256	128	−	128	−
**5**	128	−	128	256	128	−	128	−
**6**	−	−	−	−	−	−	−	−
**7a**	−	−	−	−	−	−	−	−
**7b**	−	−	−	−	−	−	−	−
**8**	32	−	−	−	64	−	−	−
**(B)**								
**Compounds**	***S. aureus* B1**		***S. aureus* B1**		***E. faecalis* W1**		***E. faecium* W5**	
	**MIC**	**MBC**	**MIC**	**MBC**	**MIC**	**MBC**	**MIC**	**MBC**
**4a**	8	−	8	−	16	−	16	−
**8**	32	−	32	−	−	−	−	−

(−): >256 µg/mL.

**Table 4:** Antibacterial efficacy (halos, mm) of combined effect of antibiotics with compounds **1**–**8** (15 µg/disc) against three multidrug-resistant isolates, using the disc diffusion method.

	*E. coli* G1				*S. aureus* B1			*E. faecium* W5		
	Antibiotics									
Compounds	CIP	AMP	CTX	S	OX	AMP	CTX	VA	AMP	E
**1a**	7	7	=	7.5	=	=	=	=	=	=
**1b**	7	7	=	7.5	=	=	=	=	=	=
**1c**	7	7	=	7.5	=	=	=	=	=	=
**1d**	7	7	=	7.5	=	=	=	=	=	=
**2**	7	7	=	7.5	=	=	=	=	=	=
**3**	7	7	=	7.5	=	=	=	=	=	=
**4a**	8	8	=	8	12.5	13	13	11	12	13.5
**4b**	7	7	=	7.5	11	10	10.5	11	12	13.5
**5**	7	7	=	7.5	=	=	=	=	=	=
**6**	7	7	=	7.5	=	9	=	=	=	=
**7a**	8	8	=	8	9.5	10	9.5	8.5	9	8
**7b**	7	7.5	=	7.5	8.5	9.5	8.5	=	=	=
**8**	7.5	7.5	=	7.5	10	10	10	8.5	7	7
**Control**	0	0	14	0	0	7	0	8	0	0

Control: Antibiotic with no compounds; CIP: Ciprofloxacin; AMP: Ampicillin; CTX: Cefotaxime; S: Streptomycin; OX: Oxacillin; VA: Vancomycin; E: Erythromycin. (=): Indicates no influence of the compound; same result as obtained with no compound.

**Table 5:** Fractional inhibitory concentration (FIC) index results obtained with **4a**/**8** and antibiotic combinations by checkerboard method.

Bacterial Isolate	4a-OX		8-OX		4a-VA		8-Va		4a-AMP		8-AMP	
	ΣFIC	Activity ^a^	ΣFIC	Activity	ΣFIC	Activity	ΣFIC	Activity	ΣFIC	Activity	ΣFIC	Activity
***S. aureus* B1**	0.562	I	0.516	**I**	−	−	−	−	2	**I**	0.516	**I**
***S. aureus* B2**	2	I	0.625	**I**	−	−	−	−	2	**I**	0.625	**I**
***E. faecalis* W1**	−	−	−	−	0.312	S	−	−	0.75	**I**	−	−
***E. faecium* W5**	−	−	−	−	0.312	S	−	−	0.75	S	−	−

AMP: Ampicillin; OX: Oxacillin; VA: Vancomycin; ^a^ S = synergism; I = indifference; (−): Not determined.

The disc diffusion method (Table 4) revealed a small synergistic association between all the compounds tested and the antibiotics to which *E. coli* G1 was resistant. Even though only a few compounds showed synergism against *S. aureus* B1 and *E. faecium* W5, association of aszonapyrone A (**4a**) with the antibiotics was found to produce the biggest halos, whereas sartorypyrone A (**8**) increased the antibiotic inhibition halos against *S. aureus* B1 and, to a lesser extent, against *E. faecium* W5. Interestingly, although chevalone C (**7a**) alone did not show antibacterial activity at the highest concentration tested (MIC > 256 mg/mL), it demonstrated a synergistic effect with antibiotics against all three multidrug-resistant isolates. The results of the Checkerboard method, represented by the FIC index, are shown in Table 5. The combination effect of aszonapyrone A (**4a**) with oxacillin (OX) and ampicillin (AMP) against MRSA and VRE isolates, respectively, was found to be indifferent (ΣFIC > 0.5); however, aszonapyrone A (**4a**) was found to lower the MIC of each antibiotic tested, thus, it may be considered a partially synergist effect. The association of aszonapyrone A (**4a**) with vancomycin (VA) showed a clear synergistic effect (ΣFIC < 0.5) against the two VRE isolates tested. The combination of sartorypyrone A (**8**) with OX and AMP against MRSA isolates was found to be also indifferent. Since the MIC of sartorypyrone A (**8**) against VRE was higher than 256 µg/mL, no checkerboard method was performed for this compound against VRE isolates.

**Figure 2:**
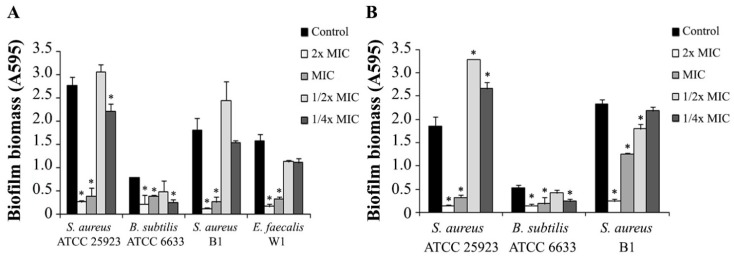
Biomass quantification of biofilms of Gram-positive bacteria formed in the presence of different concentrations (ranging from 2× MIC to 1/4× MIC) of **4a **(**A**) and **8 **(**B**).

**Figure 3:**
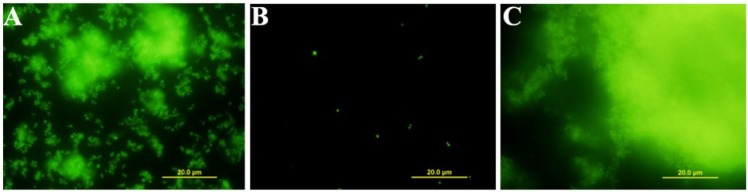
Evaluation of *S. aureus* ATCC 25953 biofilm formation. Live/dead viability staining images after 24 h. Control (**A**); Biofilm formation in the presence of the MIC (**B**) and in the presence of ½ of the MIC (**C**) of **4a**.

The effect of aszonapyrone A (**4a**) and sartorypyrone A (**8**), at different concentrations (ranging from 2× MIC to 1/4× MIC), on the biofilm formation of *S. aureus* ATCC 25923, *B. subtilis* ATCC 6633 and *S. aureus* B1, and also *E. faecalis* W1 (in the case of **4a**) was also assessed using the biomass quantification, and the results are shown in Figure 2. All the strains tested showed no biofilm formation in the presence of 2xMIC and MIC of aszonapyrone A (**4a**) and sartorypyrone A (**8**). However, *S. aureus* ATCC 25923 and *S. aureus* B1 formed more biofilm in the presence of a sub-inhibitory concentration (1/2× MIC) of aszonapyrone A (**4a**) (Figure 2A). Moreover, *S. aureus* ATCC 25923 was found to produce a significantly (*P* < 0.05) higher amount of biomass in the presence of 1/2× MIC of sartorypyrone A (**8**), when compared to the control (Figure 2B). In order to confirm the effect of these compounds on biofilm formation, the microscopic visualization of the biofilm produced by *S. aureus* ATCC 25923 was carried out using a Live/Dead staining. After 24 h, the majority of the cells within the biofilm were viable and large aggregates embedded in a matrix could be observed (Figure 3A). In the presence of aszonapyrone A (**4a**), at a concentration equal to the MIC, no biofilm was formed and also no growth was observed (Figure 3B). However, at the concentration of 1/2× MIC, it was possible to observe more biofilm in comparison to the control (Figure 3C). These results are in agreement with those obtained in the biomass quantification for the same experimental conditions. However, this result is not unexpected since there are several reports of the increase in biofilm formation in both Gram-positive and Gram-negative bacteria in the presence of sub-inhibitory concentrations of antibiotics [18,19,20]. Interestingly, the BIC value of aszonapyrone A (**4a**) was found to be higher than 12× MIC against mature biofilms of both *S. aureus* B1 (BIC > 96 µg/mL) and *E. faecalis* W1 (BIC > 192 µg/mL). However, the exact BIC value could not be determined due to the limited quantity of this compound available to perform all these biological assays. These very high BIC values may reflect the difficulty of **4a** in penetrating the extracellular biofilm matrix, thus hampering the eradication of the pre-established biofilm.

Examination of the structures of the meroditerpenes tested (Figure 1) suggested the existence of some common features necessary for the antibacterial activity of this class of compounds. Although aszonapyrone A (**4a**), aszonapyrone B (**4b**), sartorypyrone C (**5**) and sartorypyrone A (**8**), all contain the 4-hydroxy-6-methyl-2*H*-pyran-2-one ring, only aszonapyrone A (**4a**) and sartorypyrone A (**8**) have the β-acetoxyl group on C-3. On the other hand, this 4-hydroxy-6-methyl-2*H*-pyran-2-one ring is connected to the perhydrophenanthrene portion by the ethereal bridge, forming a more rigid pentacyclic structure in chevalone B (**6**). On the other contrary, both chevalone C (**7a**) and sartorypyrone B (**7b**) contain the 6-methyl-4*H*-pyran-4-one ring connected to the perhydrophenanthrene portion by an ethereal bridge. Therefore, it is apparent that the presence of a free 4-hydroxy-6-methyl-2*H*-pyran-2-one ring on C-15 and the β-acetoxyl group on C-4 of the perhydrophenanthrene portion are required for the antibacterial activity of this series of meroditerpenes.

## 3. Experimental Section

### 3.1. General Experimentation Procedures

Melting points were determined on a Bock monoscope and are uncorrected. Optical rotations were determined on an ADP410 Polarimeter (Bellingham+Stanley Ltd., Tunbridge Wells, Kent, UK) Infrared spectra were recorded on an ATT Mattson Genesis Series FTIR™ using WinFIRST Software. ^1^H and ^13^C NMR spectra were recorded at ambient temperature on a Bruker AMC instrument (Bruker Biosciences Corporation, Billerica, MA, USA) operating at 300.13 and 75.4 MHz, respectively. High resolution mass spectra were measured with a Xevo QToF mass spectrometer (Waters Corporations, Milford, MA, USA) coupled to the Aquity UPLC system (Waters Corporations, Milford, MA, USA). A Merck silica gel GF_254_ was used for preparative TLC, and a Merck Si gel 60 (0.2–0.5 mm) was used for analytical chromatography.

### 3.2. Extraction and Isolation

Extraction and Isolation of Secondary Metabolites from the Culture of *Neosartorya paulistensis* (KUFC 7897)

*Neosartorya paulistensis* (KUFC 7897) was isolated from the marine sponge *Chondrilla australiensis* which was collected from Mu Kho Lan Beach, Chonburi Province, Thailand in May 2010. After rinsing with sterile sea water, the sponge was dried on sterile filter papers and cut into small pieces (5 × 5 mm) and placed on the plates containing malt extract agar (MEA, 30 g of malt extract, 15 g of bacto agar, distilled water 1000 mL and adjusted to the final pH at 5.5) with 70% sea water and incubated at 28 °C under 12 h light/12 h dark cycle for 7 days. The fungus was identified by Prof. Dr. Leka Manoch (Department of Plant Pathology, Kasetsart University, Bangkok, Thailand), by morphological features, including the characteristic of ascospores and colonies. The identification was supported by sequence analysis of the β-tubulin gene described in the previous report [21] and the pure cultures were deposited as KUFC 7897 at Kasetsart University Fungal Collection, Department of Plant Pathology, Faculty of Agriculture, Kasetsart University, Bangkok, Thailand, and as MMERU 02 at Microbes Marine Environment Research Unit, Division of Environmental Science, Faculty of Science, Ramkhamhaeng University, Bangkok, Thailand. The fungus was cultured for two weeks at 28 °C in 10 Petri dishes (i.d. 90 mm) containing 25 mL of MEA with 70% sea water per dish. Fifty 1000 mL Erlenmeyer flasks each containing rice (200 g), water (30 mL), and sea water (70 mL), were autoclaved, inoculated with five mycelia plugs of *N. paulistensis* and incubated at 28 °C for 30 days, after which the mouldy rice was macerated in ethyl acetate (15 L total) for 10 days and then filtered. The two layers were separated using a separatory funnel and the ethyl acetate solution was concentrated at a reduced pressure to yield crude ethyl acetate extract, which was washed with 5% of NaHCO_3_ solution (2 × 500 mL) and H_2_O (3 × 500 mL). The organic layer was dried with anhydrous Na_2_SO_4_, filtered and evaporated under reduced pressure to give 51 g of crude extract which was applied on a column chromatography over 610 g of Si gel (0.2–0.5 mm, Merck KGaA, Darmstadt, Germany) and eluted with mixtures of CHCl_3_–petrol and CHCl_3_–Me_2_CO, wherein 250 mL fractions were collected as follows: fractions 1–25 (CHCl_3_–petrol, 1:4), 26–149 (CHCl_3_–petrol, 3:7), 150–177 (CHCl_3_–petrol, 2:3), 178–278 (CHCl_3_–petrol, 1:1), 279-399 (CHCl_3_–petrol, 3:2), 400–534 (CHCl_3_–petrol, 4:1), 535–549 (CHCl_3_–petrol, 9:1), 550–592 (CHCl_3_), 593–837 (CHCl_3_–Me_2_CO, 9:1), 838–976 (CHCl_3_–Me_2_CO, 8:2), 977–1047 (CHCl_3_–Me_2_CO, 7:3), 1048–1090 (CHCl_3_–Me_2_CO, 1:1) and 1091–1112 (CHCl_3_–Me_2_CO, 1:4). Frs 299–307 (526 mg) were combined and recrystallized in petrol to give 12 mg of sartorypyrone C (**5**). Frs 627–696 were combined (1.50 g) and crystallized in a mixture of CHCl_3_ and acetone to give 70 mg of tryptoquivaline L (**1a**). Frs 739–774 were combined (400 mg) and crystalized in Me_2_CO to give 95 mg of tryptoquivaline H (**1b**). The mother liquor of frs 739–774 and frs 697–774 were combined (850 mg), applied on a column chromatography of LiChroprep Si 60 (40–63 mm, 46 g) and eluted with mixtures of CHCl_3_–petrol and CHCl_3_–Me_2_CO wherein 100 mL sub-fractions were collected as follows: subfrs 1–24 (CHCl_3_–petrol, 4:1), 25–49 (CHCl_3_–petrol, 9:1), 50–63 (CHCl_3_), 64–142 (CHCl_3_–Me_2_CO, 9:1) and 143–163 (CHCl_3_–Me_2_CO, 4:1). Subfrs 82–86 were combined (31 mg) and purified by TLC (Si gel, CHCl_3_–Me_2_CO–EtOAc–HCO_2_H, 7:2:1:0.1) to give 8.3 mg of tryptoquivaline L (**1a**) and 9.6 mg of 4(3*H*)-quinazolinone (**3**) [22]. Subfrs 87–102 were combined (250 mg) and purified by TLC (Si gel, CHCl_3_–Me_2_CO–EtOAc–HCO_2_H, 7:2:1:0.1) to give 38 mg of tryptoquivaline L (**1a**) and 18 mg of tryptoquivaline H (**1b**). Subfrs 103–130 were combined (157 mg) and crystallized in Me_2_CO to give 27 mg of tryptoquivaline H (**1b**). Frs 775–832 were combined (690 mg) and crystalized in Me_2_CO to give 95 mg of 3′-(4-oxoquinazolin-3-yl)spiro[1*H*-indole-3,5′]-2,2′-dione (**2**). The mother liquor of frs 775–832 and frs 833-860 were combined (902 mg) and applied over a LiChroprep Si 60 column (40–63 µm, 41 g, Merck KGaA, Darmstadt, Germany) and eluted with mixtures of CHCl_3_–petrol and CHCl_3_–Me_2_CO wherein 100 ml sub-fractions were collected as follows: subfrs 1–26 (CHCl_3_–petrol, 4:1), 27–41 (CHCl_3_), 42–108 (CHCl_3_–Me_2_CO, 9:1). Subfrs 61–68 were combined and purified by TLC (Si gel, CHCl_3_–Me_2_CO–EtOAc–HCO_2_H, 7:2:1:0.1) to give 4.2 mg of tryptoquivaline L (**1a**) and 18 mg of tryptoquivaline H (**1b**). Subfrs 69-86 were combined (176 mg) and recrystallized in Me_2_CO to give 15 mg of 3′-(4-oxoquinazolin-3-yl)spiro[1*H*-indole-3,5′]-2,2′-dione (**2**). Frs 905–947 were combined (670 mg) and crystalized in Me_2_CO to give 16.4 mg of tryptoquivaline F (**1c**). The structures of the compounds were established by comparison their NMR spectral data with those in the literature [16] as well as with authentic samples.

#### 3.2.1. Sartorypyrone C (**5**)

White solid, Mp = 200–202 °C. [α]_D_^25^ = −73.5° (*c* 0.07, CHCl_3_); IR (KBr) ν_max_ 3445, 2923, 2942, 2853, 1699, 1668, 1649, 1636, 1507, 1124 cm^−1^. ^1^H and ^13^C see Table 1. HR-ESIMS *m/z* 415.2836 (M + H)^+^ (calculated for C_28_H_41_O_5_, 415.2848).

#### 3.2.2. Isolation of Tryptoquivaline T (**1*d***) from the Culture of *Neosartorya laciniosa* (KUFC 7896)

Extraction and isolation of tryptoquivaline L (**1a**) 3′-(4-oxoquinazolin-3-yl) spiro[1*H*-indole-3,5′]-2,2′-dione (**2**), aszonapyrone A (**4a**) and aszonapyrone B (**4b**) from the culture of the diseased coral derived fungus *Neosartorya laciniosa* (KUFC 7896) have been previously described by us [15]. The column fractions 498–507 (793 mg) and mother liquor of the combined frs 508–546 (1.57 g) were combined and chromatographed over a LiChroprep Si 60 column (40–63 µm, 36 g, Merck KGaA, Darmstadt, Germany) and eluted with mixtures of CHCl_3_–petrol and CHCl_3_–Me_2_CO wherein 100 mL sub-fractions were collected as follows: Subfrs 1–84 (CHCl_3_–petrol, 9:1), 85–137 (CHCl_3_–Me_2_CO, 9:1), 138–162 (CHCl_3_–Me_2_CO, 4:1). Subfrs 43–50 were combined (214 mg) and recrystallized in Me_2_CO to give 13 mg of tryptoquivaline T (**1d**).

##### Tryptoquivaline T (**1d**)

White solid, Mp = 258–260 °C. [α]_D_^25^ = −83.3 (*c* 0.04, MeOH); IR (KBr) ν_max_ 3010, 2930, 2852, 1701, 1582, 1450, 1402, 1269 cm^−1^. ^1^H and ^13^C see Table 2. HR-ESIMS *m/z* 445.1512 (M + H)^+^ (calculated for C_28_H_41_O_5_, 445.1512).

#### 3.2.3. Isolation of Chevalone B (**6**) and Chevalone C (**7*a***) from the Culture of *Neosartorya siamensis* (KUFC 6349)

Isolation and identification of the fungus as well as fractionation of the crude extract and isolation of other constituents from the culture of *Neosartorya siamensis* (KUFC 6349) have been previously described by us [16]. Fr 34 (1.02 g) was applied on a column chromatography of Si gel (35 g) and eluted with mixtures of petrol–CHCl_3_ and CHCl_3_–Me_2_CO, 100 mL fractions were collected as follows: Subfrs 1–32 (CHCl_3_–petrol 3:7), 33–54 (CHCl_3_–petrol, 1:1), 55–58 (CHCl_3_–petrol, 7:3). Subfrs 15–17 were combined (0.28 g) and crystallized in mixture of petrol and CHCl_3 _to give 137.5 mg of chevalone B (**6**). Frs 35 (630 g) was applied on a column chromatography of Si gel (35 g) and eluted with mixtures of petrol–CHCl_3_, 100 mL fractions were collected as follows: Frs 1–52 (CHCl_3_–petrol, 3:7), 53–64 (CHCl_3_–petrol, 1:1). Subfrs 33–52 were combined (63.6 mg) and purified by TLC (Si Gel, CHCl_3_–Me_2_CO–HCO_2_H, 95:0.5:0.1) to give 12 mg of chevalone C (**7a**). Subfrs 53–56 were combined (300 mg) and crystallized in petrol to give 40.3 mg of chevalone C (**7a**). Frs 36–53 were combined (1.80 g) and crystallized in a mixture of petrol and CHCl_3_ to give 375 mg of chevalone C (**7a**). Frs 83–100 were combined (300 mg) and crystallized in a mixture petrol and CHCl_3_ to give 112.5 mg of chevalone C (**7a**).

### 3.3. Antibacterial Activity Bioassays

#### 3.3.1. Bacterial Strains

For the antibacterial bioassays, fungal metabolites were tested against reference strains, two Gram-positive (*Staphylococcus aureus* ATCC 25923 and *Bacillus subtilis* ATCC 6633) and two Gram-negative (*Escherichia coli* ATCC 25922 and *Pseudomonas aeruginosa* ATCC 27853) bacteria, and against multidrug-resistant isolates from the environment, *S. aureus* B1 and *S. aureus* B2 (isolated from public buses), *Enterococcus faecalis* W1 and *E. faecium* W5 (isolated from river water) and *E. coli* G1 (isolated from seagull feces). These bacteria were grown in Mueller-Hinton agar (MH—BioKar diagnostics, Allonne, France) from stock cultures. MH plates were incubated at 37 °C prior to obtain fresh cultures for each *in vitro* bioassay.

#### 3.3.2. Determination of Minimum Inhibitory and Bactericidal Concentrations

The minimum inhibitory concentration (MIC) values of the 13 compounds were determined by a broth microdilution technique, following the recommendations of the Clinical and Laboratory Standards Institute [23]. Stock solutions of 10 mg/mL prepared by dissolving each metabolite in dimethylsulfoxide (DMSO—Applichem GmbH, Darmstadt, Germany) were serial diluted in Mueller-Hinton broth (MHB—BioKar diagnostics, Allonne, France) to achieve in-test concentrations ranging from 2 to 256 µg/mL. Ciprofloxacin in the concentration range from 0.03125 to 16 μg/mL was used as control drug in the experiment. A bacterial inoculum was prepared in MHB and standardized in order obtain a concentration of 5 × 10^5^ CFU/mL in each inoculated well of the microtiter plate. The concentration of DMSO in the highest in-test concentration did not affect the microbial growth. The MIC was defined as the lowest concentration of the compound that inhibited the visible growth. The minimum bactericidal concentration (MBC) was determined by spreading 10 µL on MH plates from the sample showing no visible growth and it was further incubated for 24 h at 37 °C; the lowest concentration at which no bacterial growth occurred on MB plates was defined as the MBC.

#### 3.3.3. Synergistic Studies

A screening susceptibility test to assess combined effect between compounds **1**–**8** and antibiotics was conducted using the disc diffusion method on MH. Multidrug-resistant isolates were picked from overnight cultures in MH, and suspensions were prepared in buffered peptone water (Oxoid, Basingstoke, England) by adjusting the turbidity to equal a 0.5 McFarland standard. A set of antibiotic discs (Oxoid, Basingstoke, England) was selected based on the resistance of the isolates towards those antibiotics. Antibiotic discs alone (controls) and impregnated with 15 µL of a 1 mg/mL solution (in DMSO) of each metabolite were placed on the agar plate seeded with the respective bacteria. Fifteen µL of DMSO impregnated in a sterile filter paper disc (6 mm in diameter) (Oxoid, Basingstoke, England) was used as the negative control. Inoculated MH plates were incubated overnight at 37 °C. Each compound was tested in duplicate. Potential synergism was recorded when the halo of antibiotic discs impregnated with metabolites was greater than the halo of antibiotic discs or compound-impregnated blank discs alone. 

Based on the results of the previous assay, potential synergism between the most promising compounds (**4a** and **8**) and antibiotics (oxacillin, vancomycin and ampicillin—Sigma-Aldrich, St. Louis, MO, USA) was checked using a broth microdilution checkerboard method and tested against *S. aureus* MRSA and *Enterococcus* spp. VRE isolates. Briefly, the stock solutions and serial twofold dilutions of each compound and antibiotic to at least double the MIC were prepared according to the recommendations of CLSI [23]. The metabolite to be tested was serially diluted along the ordinate, while the antibiotic was diluted along the abscissa. A bacterial inoculum equal to a 0.5 McFarland turbidity standard was prepared in MHB. Each microtiter plate well was inoculated with 100 µL of a bacterial inoculum of 5 × 10^5^ CFU/mL, and the plates were incubated overnight at 37 °C. The fractional inhibitory concentration (FIC) was calculated as follows: FIC of drug A (FIC A) = MIC of drug A in combination/MIC of drug A alone, and FIC of drug B (FIC B) = MIC of drug B in combination/MIC of drug B alone. The FIC index (ΣFIC), calculated as the sum of each FIC, was interpreted as follows: ΣFIC ≤ 0.5, synergy; 0.5 < ΣFIC ≤ 4, no interaction; 4 < ΣFIC, antagonism [24].

#### 3.3.4. Antibiofilm Activity Assay

Given the promising antibacterial activity of **4a** and **8** against Gram-positive bacteria, the efficacy of those two compounds in interrupting the biofilm formation was assessed. The metabolites at concentrations of 2× MIC, MIC, 1/2× MIC and 1/4× MIC were added to bacterial suspensions of 1 × 10^6^ CFU/ml in Tryptic Soy broth (TSB—BioKar diagnostics, Allonne, France). Bacterial suspension without metabolites was used as the control. Each broth culture obtained was dispensed into a 96-well microtiter plate (200 µL/well) and incubated at 37 °C for 24 h. After that time, biofilm was stained with 0.5% crystal violet for 5 min, rinsed with water, air dried and eluted with acetic acid 33% (v/v). The optical density was measured at 595 nm (OD_595_) using a microplate reader (iMark™ microplate absorbance reader, Bio-Rad Laboratories, Hercules, CA, USA). Two independent experiments were performed in triplicate for each experimental condition. The statistical significance of difference between biofilms of controls and biofilms in the presence of different concentrations of compounds was evaluated using Student’s t test. In both cases, probability levels <0.05 were considered statistically significant. The efficacy of **4a** on established biofilm of *S. aureus* B1 and *E. faecalis* W1 was also evaluated by determining the biofilm inhibitory concentration (BIC) according to the method described by Johnson *et al.* [25]. Briefly, bacterial suspensions in TSB at 1 × 10^6^ CFU/mL were used to grow the biofilms in 96-well microtiter plate. After 24 h of incubation at 37 °C, the planktonic cells were gently removed and the wells were rinsed once and filled with different dilutions ranging from the MIC values to 12× MIC. The OD_595_ was measured at time 0 and after incubation for 24 h at 37 °C. The BIC was determined as the lowest concentration of the compound inhibiting growth in the supernatant fluid, confirmed by no increase in optical density compared with the initial reading.

Additionally, microscopic visualization of biofilms of *S. aureus* ATCC 25923 was performed using the Live/Dead BacLight viability kit (Life Technologies—Molecular Probes, Carlsbad, CA, USA). Biofilms were formed in 35-mm diameter polystyrene plates using TSB (control) and TSB supplemented with MIC and 1/2× MIC of **4a**. After 24 h at 37 °C. The planktonic phase was removed from each plate, washed with PBS, stained with the appropriate mixture of SYTO 9 and propidium iodide stains and incubated for 20 minutes at room temperature in the dark; then, were rinsed and examined under a fluorescence microscope (BX41 Microscope, Olympus America Inc., Center Valley, PA, USA). Images were recorded at an emission wavelength of 500 nm for SYTO 9 (green fluorescence) and of 635 nm for propidium iodide (red fluorescence).

## 4. Conclusions

We reported the isolation and structure elucidation of a new meroditerpene, sartorypyrone C (**5**), together with the previously described tryptoquivalines L (**1a**), H (**1b**), F (**1c**), 3′-(4-oxoquinazolin-3-yl) spiro[1*H*-indole-3,5′]-2,2′-dione (**2**) and 4(3*H*)-quinazolinone (**3**), from the culture of the marine sponge-associated fungus *N. paulistensis* (KUFC 7897), as well as a new tryptoquivaline derivative which we have named tryptoquivaline T (**1d**), from the culture of the diseased coral-derived fungus *N. laciniosa* (KUFC 7896). Antibacterial activity evaluation of the isolated compounds, together with the previously described meroditerpenes aszonapyrones A (**4a**) and B (**4b**), chevalones B (**6**) and C (**7a**), sartorypyrones B (**7b**) and A (**8**), against four reference strains (*S. aureus*, *B. subtilis*, *E. coli*, and *P. aeruginosa*), as well as the environmental multidrug-resistant isolates, showed that only the meroditerpenes aszonapyrone A (**4a**) and sartorypyrone A (**8**) were active against both Gram-negative reference strains and the environmental multidrug-resistant isolates. Aszonapyrone A (**4a**) also exhibited a clear synergistic effect with vancomycin against the two VRE isolates, while it showed only partially synergist effect with oxacillin and ampicillin against MRSA and VRE isolates. Both aszonapyrone A (**4a**) and sartorypyrone A (**8**) were found to inhibit the biofilm formation in both reference strains (*S. aureus* ATCC 25923 and *B. subtilis* ATCC 6633) and the multi-drug resistant isolates (*S. aureus* B1 and *E. faecalis* W1), at the 2× MIC and MIC.

In light of the results presented in this work, we can conclude that the meroditerpenes aszonapyrone A (**4a**) and sartorypyrone A (**8**) are two promising antibacterial agents, especially against Gram-positive bacteria, and their effects are comparable to those of standard antibiotics currently in use in therapeutics, with the advantage of being also active against bacteria that already exhibit resistance towards these antibiotics. To the best of our knowledge, none of the available antibiotics belongs to this class of compounds, which thereby may represent a novel and potential topic of investigation in the field of new antibacterial agents from the marine-derived fungi. 

## Supplementary Files

Supplementary File 1 Supplementary Information (PDF, 471 KB)

## References

[1] Johnson A.P., Livermore D.M., Tillotson G.S. (2001). Antimicrobial susceptibility of Gram-positive bacteria: What’s current, what’s anticipated?. J. Hosp. Infect..

[2] Aksoy D.Y., Unal S. (2008). New antimicrobial agents for treatment of Gram-positive bacterial infections. Clin. Microbiol. Infect..

[3] Aziz A.M. (2013). The role of healthcare strategies in controlling antibiotic resistance. Br. J. Nurs..

[4] Bassetti M., Merelli M., Temperoni C., Astilean A. (2013). New antibiotics for bad bugs: Where are we?. Ann. Clin. Microbiol. Antimicrob..

[5] Kijjoa A., Sawangwong P. (2004). Drugs and Cosmetics from the Sea. Mar. Drugs.

[6] Newman D.J., Cragg G.M. (2012). Natural Products as Sources of New Drugs over the 30 Years from 1981 to 2010. J. Nat. Prod..

[7] Blunt J.W., Copp B.R., Keyzers R.A., Munro M.H.G., Prinsep M.R. (2013). Marine natural products. Nat. Prod. Rep..

[8] Rowley D.C., Kelly S., Kauffman C.A., Jensen P.R., Fenical W. (2003). Halovirs A–E, new antiviral agents from a marine-derived fungus of the genus *Scytalidium*. Bioorg. Med. Chem..

[9] Shen S., Li W., Ouyang M.A., Wu Z.J., Lin Q.Y., Xie L.H. (2009). Identification of two marine fungi and evaluation of their antivirus and antitumor activities. Acta Microbiol. Sin..

[10] Bugni T.S., Ireland C.M. (2004). Marine-derived fungi: A chemically and biologically diverse group of microorganisms. Nat. Prod. Rep..

[11] Pejin B., Jovanović K.K., Mojović M., Savić A.G. (2013). New and highly potent antitumor natural products from marine-derived fungi: Covering the period from 2003 to 2012. Curr. Top. Med. Chem..

[12] Christophersen C., Crescente C., Frisvad J.C., Gram L., Nielsen J., Nielsen P.H., Rahbæk L. (1999). Antibacterial activity of marine-derived fungi. Mycopathologia.

[13] Arasu M.V., Duraipandiyan V., Ignacimuthu S. (2013). Antibacterial and antifungal activities of polyketide metabolite from marine *Streptomyces* sp. AP-123 and its cytotoxic effect. Chemosphere.

[14] Manimegalai K., Devi N.K.A., Padmavathy S. (2013). Marine fungi as a source of secondary metabolites of antibiotics. Int. J. Biotechnol. Bioeng. Res..

[15] Eamvijarn A., Gomes N.M., Dethoup T., Buaruang J., Manoch L., Silva A., Pedro M., Marini I., Roussis V., Kijjoa A. (2013). Bioactive meroditerpenes and indole alkaloids from the soil fungus *Neosartorya fischeri* (KUFC 6344), and the marine-derived fungi *Neosartorya laciniosa* (KUFC 7896) and *Neosartorya tsunodae* (KUFC 9213). Tetrahedron.

[16] Buttachon S., Chandrapatya A., Manoch L., Silva A., Gales L., Bruyére C., Kiss R., Kijjoa A. (2012). Sartorymensin, a new indole alkaloid, and new analogues of tryptoquivaline and fiscalins produced by *Neosartorya siamensis* (KUFC 6349). Tetrahedron.

[17] Mahato S.B., Kundu A.P. (1994). 13C NMR spectra of pentacyclic triterpenoids—a compilation and some salient feature. Phytochemistry.

[18] Hoffman L.R., D’Argenio D.A., MacCoss M.J., Zhang Z., Jones R.A., Miller S.I. (2005). Aminoglycoside antibiotics induce bacterial biofilm formation. Nature.

[19] Kaplan J.B., Jabbouri S., Sadovskaya I. (2011). Extracellular DNA-dependent biofilm formation by *Staphylococcus epidermidis* RP62A in response to subminimal inhibitory concentrations of antibiotics. Res. Microbiol..

[20] Bessa L.J., Grande R., Di Iorio D., Di Giulio M., Di Campli E., Cellini L. (2013). *Helicobacter pylori* free-living and biofilm modes of growth: Behavior in response to different culture media. APMIS.

[21] Glass N.L., Donaldson G.C. (1995). Development of primer sets designed for use with the PCR to amplify conserved genes from filamentous ascomycetes. Appl. Environ. Microbiol..

[22] Kulkarni S.S., Singh S., Shah J.R., Low W. K., Talele T.T. (2012). Synthesis and SAR optimization of quinazolin-4(3*H*)-ones as poly(ADP-ribose)polymerase-1 inhibitors. Eur. J. Med. Chem..

[23] Franklin R., Cockerill M.D. (2011). Performance Standards for Antimicrobial Susceptibility Testing, Twenty-First Informational Supplement M100-S21.

[24] Odds F.C. (2003). Synergy, antagonism, and what the chequerboard puts between them. J. Antimicrob. Chemother..

[25] Johnson S.A., Goddard P.A., Iliffe C., Timmins B., Rickard A.H., Robson G., Handley P.S. (2002). Comparative susceptibility of resident and transient hand bacteria to para-chloro-meta-xylenol and triclosan. J. Appl. Microbiol..

