# Supplementary material for: Antibacterial and Antibiofilm Activities of Tryptoquivalines and Meroditerpenes Isolated from the Marine-Derived Fungi Neosartorya paulistensis, N. laciniosa, N. tsunodae, and the Soil Fungi N. fischeri and N. siamensis
Source: Mar Drugs. 2014 Jan 28;12(2):822–39. doi: 10.3390/md12020822 (PMC3944517; doi:10.3390/md12020822)
Supplement: Supplementary File 1 — Supplementary Information (PDF, 471 KB) [file marinedrugs-12-00822-s001.pdf]

Supplementary Information

Figure S1. ^{1}H NMR spectrum of compound **1d** (DMSO, 300.13 MHz).
Figure S2. ^{13}C NMR spectrum of compound **1d** (DMSO, 75.47 MHz).
Figure S3. ^{1}H NMR spectrum of compound **5** ($CDCl_3$, 300.13 MHz).
Figure S4. ^{13}H NMR spectrum of compound **5** ($CDCl_3$, 75.47 MHz).

Figure S1. ^{1}H NMR spectrum of compound **1d** (DMSO, 300.13 MHz).

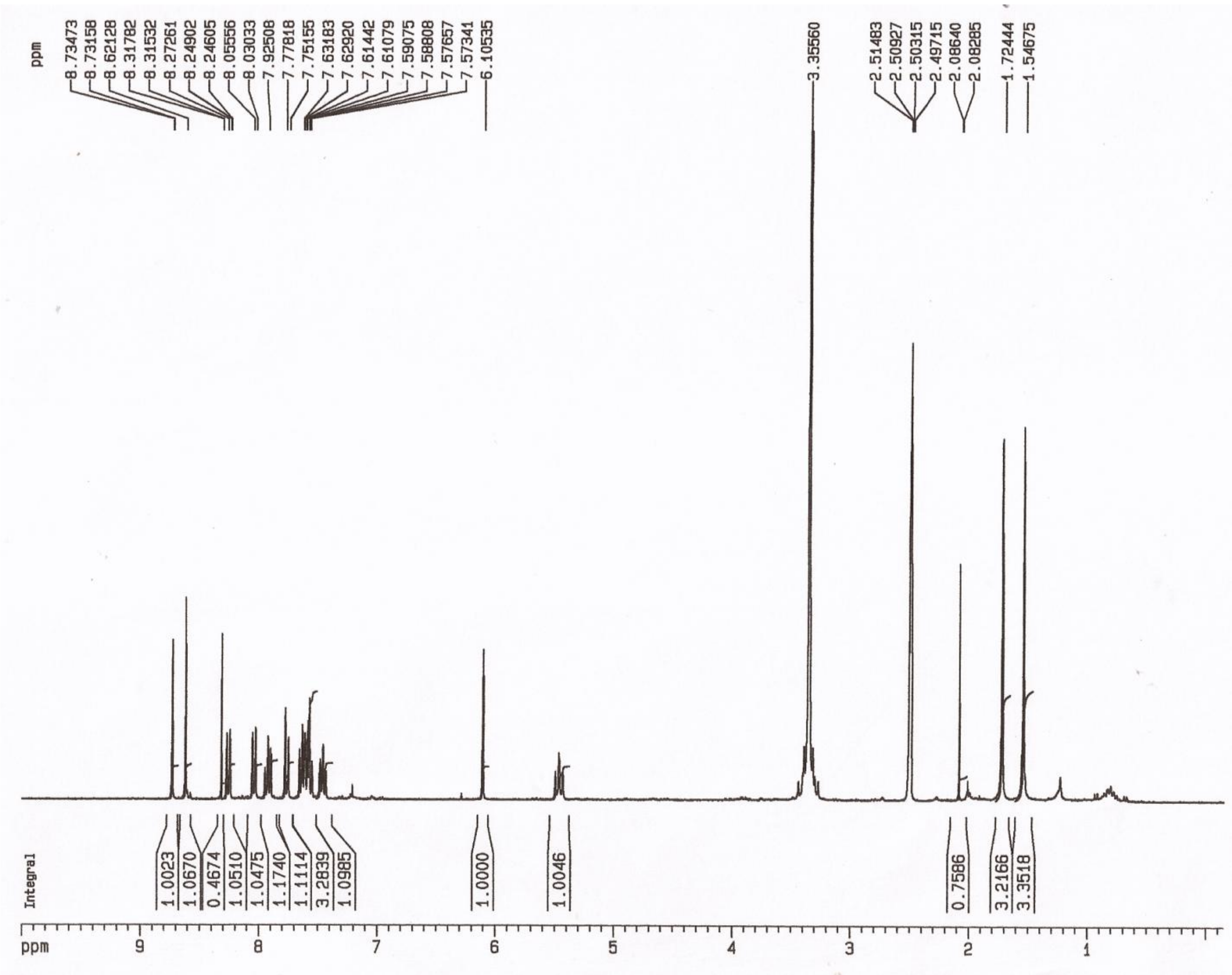

Figure S2. ^{13}C NMR spectrum of compound **1d** (DMSO, 75.47 MHz).

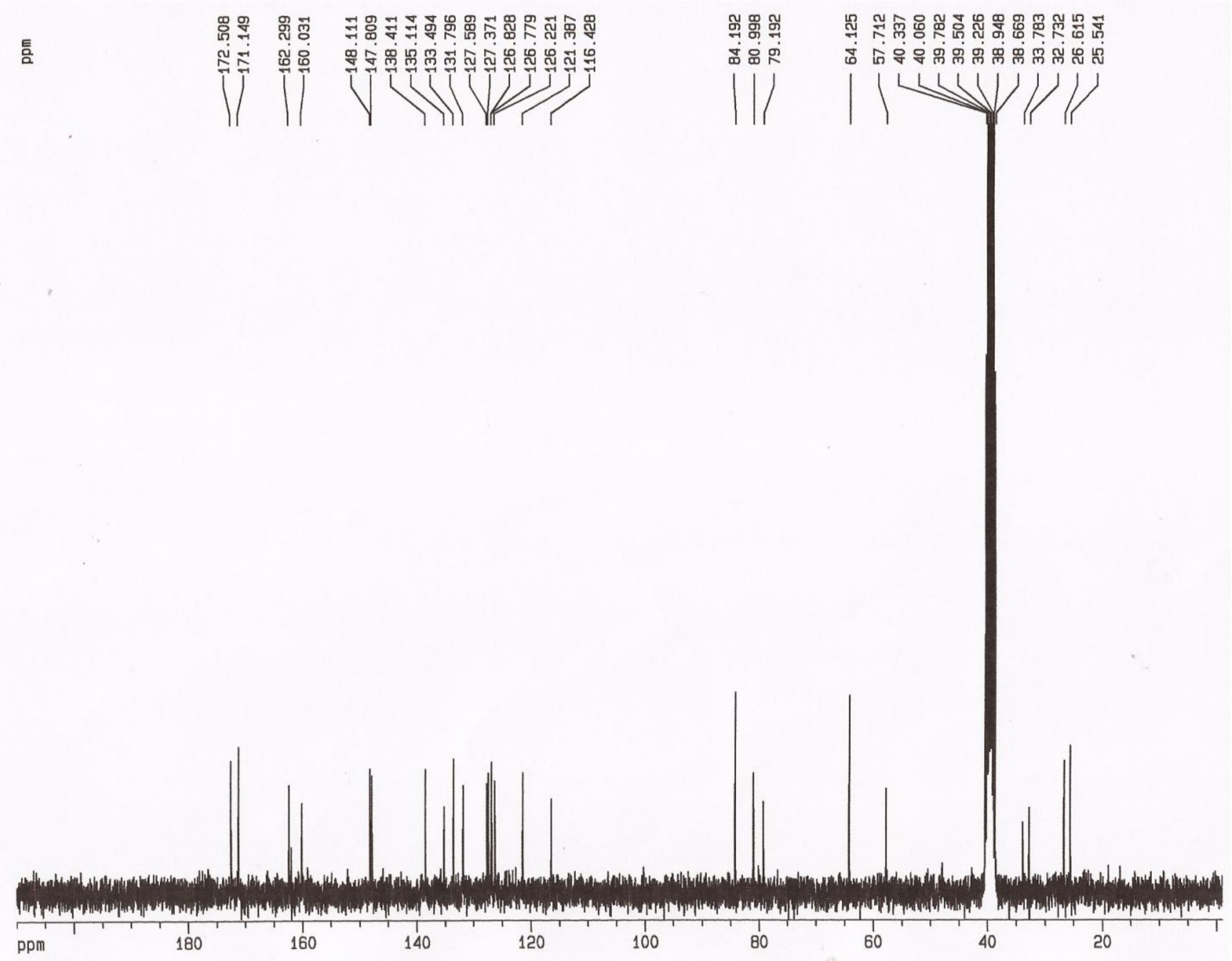

Figure S3. ^{1}H NMR spectrum of compound **5** ($CDCl_3$, 300.13 MHz).

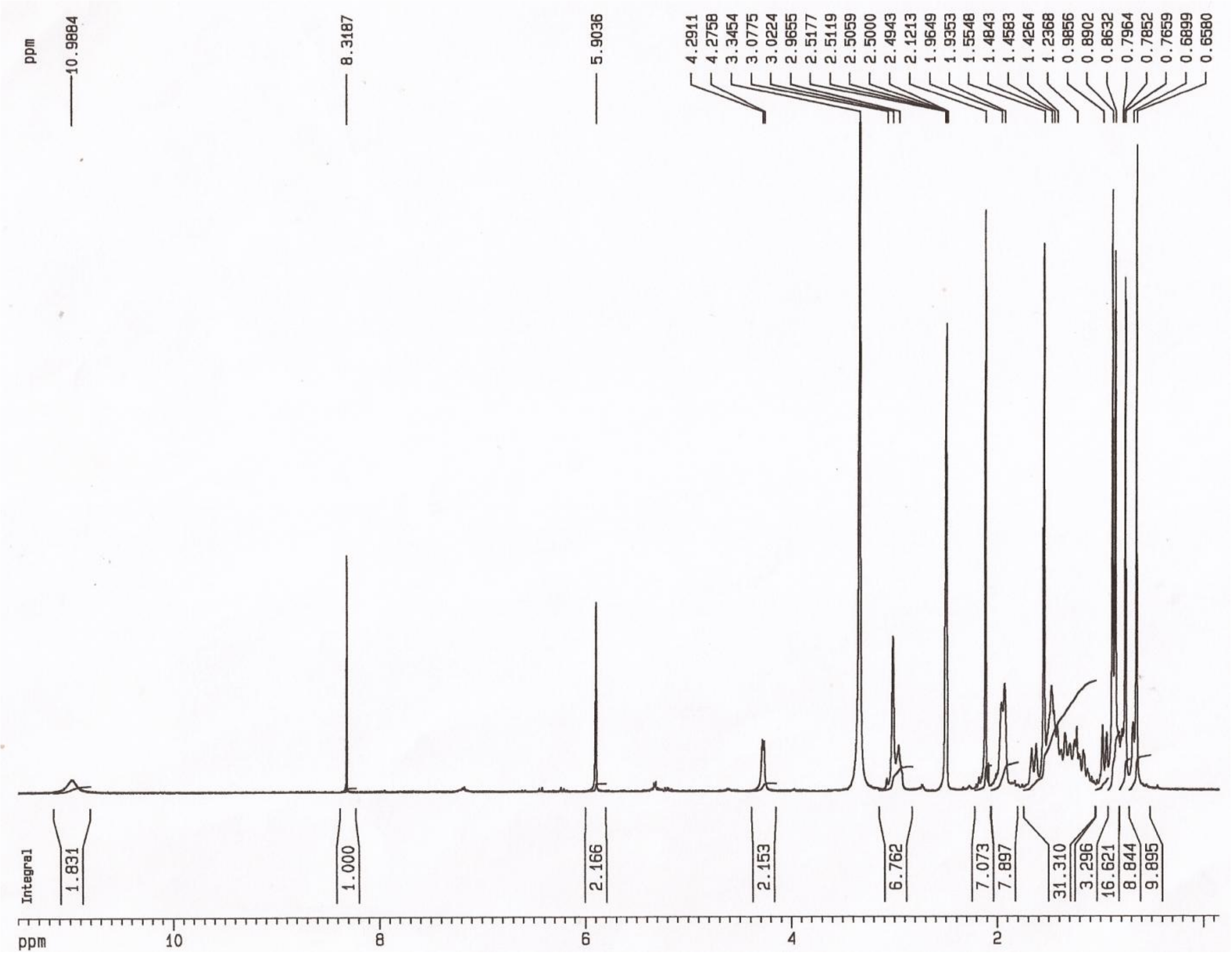

Figure S4. ^{13}H NMR spectrum of compound **5** ($CDCl_3$, 75.47 MHz).

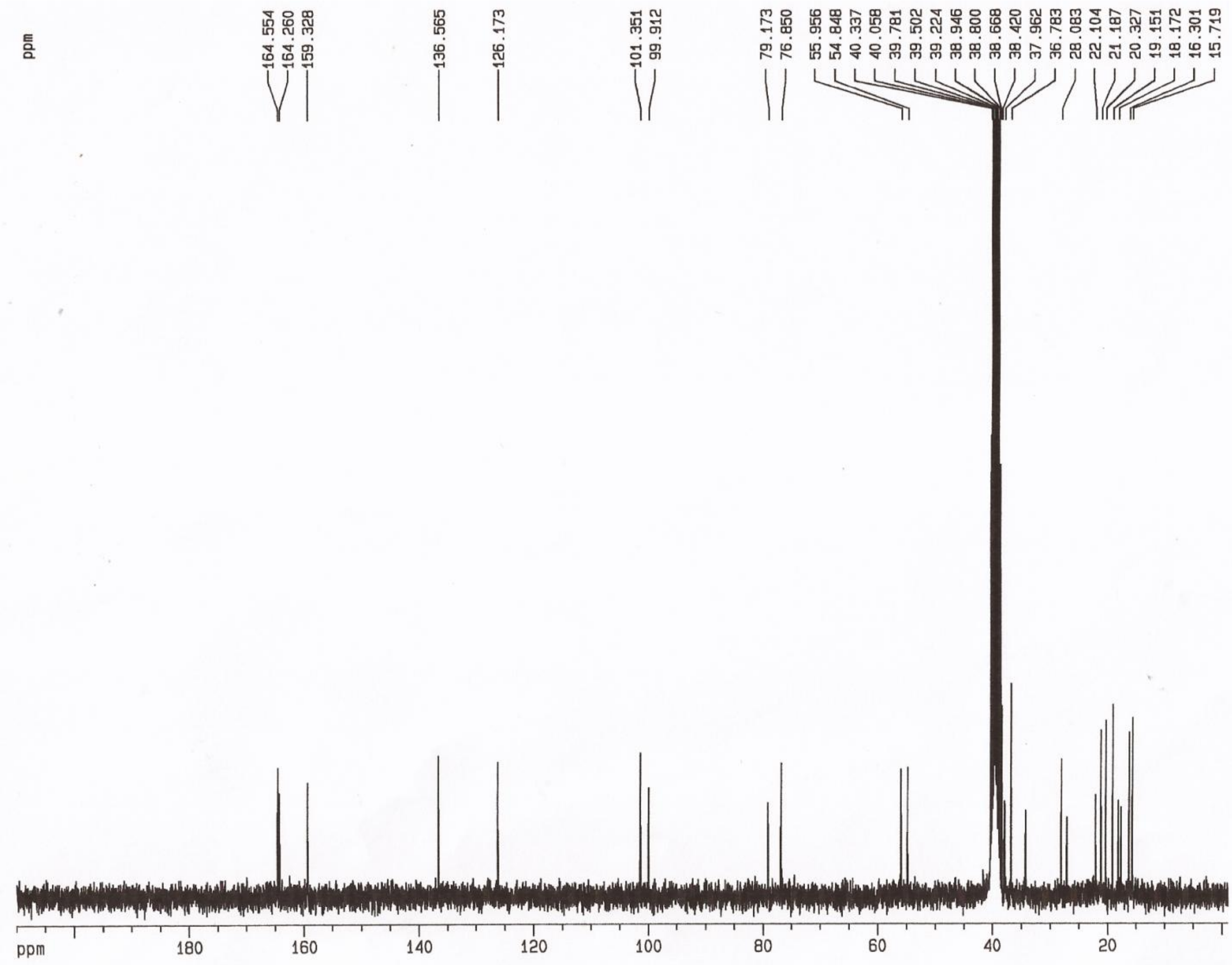

© 2014 by the authors; licensee MDPI, Basel, Switzerland. This article is an open access article distributed under the terms and conditions of the Creative Commons Attribution license (http://creativecommons.org/licenses/by/3.0/).
